# Automating risk of bias assessment in systematic reviews: a real-time mixed methods comparison of human researchers to a machine learning system

**DOI:** 10.1186/s12874-022-01649-y

**Published:** 2022-06-08

**Authors:** Patricia Sofia Jacobsen Jardim, Christopher James Rose, Heather Melanie Ames, Jose Francisco Meneses Echavez, Stijn Van de Velde, Ashley Elizabeth Muller

**Affiliations:** 1 Division for Health Services, Norwegian Institute of Public Health, Postboks 222 Skøyen, 0213 Oslo, Norway; 2 Facultad de Cultura Física, Deporte y Recreación, Cra. 9 #51-11, Bogotá, Colombia

**Keywords:** Risk of bias, Machine learning, Automation, Evidence synthesis, Systematic review, Heath technology assessment, RobotReviewer

## Abstract

**Background:**

Machine learning and automation are increasingly used to make the evidence synthesis process faster and more responsive to policymakers’ needs. In systematic reviews of randomized controlled trials (RCTs), risk of bias assessment is a resource-intensive task that typically requires two trained reviewers. One function of RobotReviewer, an off-the-shelf machine learning system, is an automated risk of bias assessment.

**Methods:**

We assessed the feasibility of adopting RobotReviewer within a national public health institute using a randomized, real-time, user-centered study. The study included 26 RCTs and six reviewers from two projects examining health and social interventions. We randomized these studies to one of two RobotReviewer platforms. We operationalized feasibility as accuracy, time use, and reviewer acceptability. We measured accuracy by the number of corrections made by human reviewers (either to automated assessments or another human reviewer’s assessments). We explored acceptability through group discussions and individual email responses after presenting the quantitative results.

**Results:**

Reviewers were equally likely to accept judgment by RobotReviewer as each other’s judgement during the consensus process when measured dichotomously; risk ratio 1.02 (95% CI 0.92 to 1.13; *p* = 0.33). We were not able to compare time use. The acceptability of the program by researchers was mixed. Less experienced reviewers were generally more positive, and they saw more benefits and were able to use the tool more flexibly. Reviewers positioned human input and human-to-human interaction as superior to even a semi-automation of this process.

**Conclusion:**

Despite being presented with evidence of RobotReviewer’s equal performance to humans, participating reviewers were not interested in modifying standard procedures to include automation. If further studies confirm equal accuracy and reduced time compared to manual practices, we suggest that the benefits of RobotReviewer may support its future implementation as one of two assessors, despite reviewer ambivalence. Future research should study barriers to adopting automated tools and how highly educated and experienced researchers can adapt to a job market that is increasingly challenged by new technologies.

**Supplementary Information:**

The online version contains supplementary material available at 10.1186/s12874-022-01649-y.

## Introduction

### Evidence synthesis and machine learning

Evidence synthesis is a detailed, resource-intensive process, aiming to collect and summarize all available evidence on a topic to produce trustworthy summaries of findings about treatment effects or syntheses of patient experiences. It has been estimated that the average health or medical-related systematic review takes more than thirteen months to complete [1]. Similarly, Shojana et al. [2] estimated that 25 % of reviews are outdated within two years of publication due to new findings.

Evidence synthesis products provide the foundation for evidence-based policy making. However, the speed at which policymakers are requesting evidence is faster than the traditional review approach can produce it. Westgate and colleagues [3] have termed this the “synthesis gap”.

Machine learning (ML) and automation techniques are rapidly developing methods that aim to reduce the resources and time required to produce an evidence synthesis and help close the synthesis gap. Unsupervised ML has recently been used to reduce time spent categorizing studies by 33% [4] and to assess the relevance of the typically thousands of records that must be screened [5]. Supervised techniques, such as an ML system designed to classify studies as randomized controlled trials (RCTs) versus other study designs, have sufficient precision and recall to be used to help produce Cochrane systematic reviews [6]. Even relatively simple ML tools can deliver meaningful benefits. For example, a text mining system in which an algorithm iteratively updates the order in which studies are presented to human reviewers for preliminary assessment, has been shown to reduce time spent on initial screening by 60–90% compared to manual sorting [4, 7, 8].

ML does not necessarily require the replacement of human effort. Rather, ML is better thought of as a way of reducing the need for humans to perform complex but repetitive tasks. Potential benefits include decreasing the time between review commission and delivery, decreased costs, less inter-review variability, and more free time for researchers to perform tasks that computers cannot.

### Assessing risk of bias

A key element of a systematic review of treatment or intervention effect is the thorough assessment of included studies’ risks of bias, or systematic errors in results or inferences [9]. For systematic reviews of randomized controlled studies, one of the most widely used tools is the Cochrane Collaboration’s tool for assessing risk of bias [9], hereafter referred to as the “RoB1” tool. This tool prompts researchers to assess five common sources, or domains, of bias, using seven questions: selection bias, performance bias, detection bias, attrition bias, reporting bias, and any other bias not covered elsewhere. Researchers assess the risk of bias within each question as *low, high*, or *unclear*, and provide a text snippet or other explanation as a support for each item’s assessment.

Risk of bias assessment requires judgement and numerous studies have reported low inter-rater reliability of the RoB1 tool [10, 11]. In practice, low inter-rater reliability means that the same RCT or RCTs may be assessed as having different levels of bias across research groups, making it possible that the same data could be translated into different recommendations [12]. However, intensive standardized training on risk of bias assessment may significantly improve the reliability of the RoB1 tool [12]. Following this logic, using technology that supports more standardized assessments might help to increase inter-rater reliability.

RobotReviewer was developed by Marshall and colleagues in 2015 [13] to automate as much of this process as possible, including the extraction of data used to support assessments and describe the RCT. Briefly, RobotReviewer performs natural language processing of text in an uploaded Portable Document Format (PDF) file of a trial. Linear models and convolutional neural networks are combined in a novel strategy to classify a document as *low* or *unclear/high* risk of bias (i.e., two categories of bias), for each of the first four questions of the Cochrane RoB1 tool: random sequence generation, allocation concealment, blinding of participants and personnel, and blinding of outcome assessors [13, 14]. RobotReviewer has been trained on 12,808 trial PDFs using data from the Cochrane Database of Systematic Reviews (CDSR) [13]. During development it exceeded the accuracy of new human assessors by 1–2% across questions, when both RobotReviewer assessments and new human re-assessments were compared to the original dataset [15]. Developers suggest that one human researcher should review RobotReviewer assessments and accept or amend them [14]. In a recent simulated validation study, volunteer reviewers agreed with 91% of RobotReviewer’s assessments, and RoB1 assessment by one reviewer with RobotReviewer was 25% faster than fully manual assessments [16].

### Context of this study

This study was conducted as part of a strategic effort to evaluate and scale up the use of ML within evidence synthesis production at the Division for Health Services at the Norwegian Institute of Public Health (NIPH). The division is staffed by 50–60 employees and typically produces approximately 35–50 evidence synthesis products per year; this has roughly doubled under COVID-19. Based on early successful implementation with ML in COVID-19 reviews [4, 17–19], a dedicated ML team was created in December of 2020 and tasked them with evaluating the time saving potential of existing ML functions, implementing those deemed successful, and horizon-scanning for new functions and applications. The team consisted of five systematic reviewers, one statistician, and one information specialist, with 3–10 years’ experience in evidence synthesis, and as of 2022, has grown to seven members and with a parallel informational specialist team [20].

### Knowledge gaps

To our knowledge, RobotReviewer has not been evaluated in real-time systematic reviews with respect to accuracy, time use, and reviewer acceptability. Arno et al.’s research [21] suggests that acceptance of ML within evidence synthesis is highly influenced by perceived methodological rigor.

The Cluster of Reviews and Health Technology Assessments at NIPH has almost two decades of experience of providing high-quality evidence syntheses, with a focus on methodological development [4, 22–26]. At NIPH, risk of bias assessments are conducted by two researchers independently of one another, who then meet to come to a consensus, that is, to decide whose assessment per question the study will ultimately receive.

As we did not know how acceptable and feasible it would be to integrate RobotReviewer into our division, we performed a real-time mixed-methods comparison of RobotReview to human reviewers.

This study aimed to assess the feasibility (accuracy, resource use, and acceptance) of RobotReviewer in two ongoing systematic reviews.

## Methods

We had the following research questions:

1. Is RobotReviewer accurate in real time assessments of the risk of bias questions in Cochrane’s RoB1 tool, when compared to the gold standard of two reviewers, in ongoing reviews?
2. Does RobotReviewer save time compared to a manual risk of bias assessment?
3. Is RobotReviewer acceptable to researchers?

### Procedures and study design

This randomized, real-time, user-centred study had two arms, each comparing two different automated procedures. The original intention was to compare any type of automation with fully manual procedures, and project leaders of both reviews were presented with two possible automated procedures and a manual risk of bias assessment. Neither project leader was interested in involving fully manual assessment.

As a result of this we chose to compare two different automated risk of bias procedures. Each arm corresponded to a different RobotReviewer platform. Both used semi-automated, human-in-the-loop risk of bias assessment procedures with RobotReviewer. In both reviews, one researcher conducted the risk of bias assessment as “first reviewer”, followed by a second reviewer who assessed as “second reviewer”. In the first arm, RR-EPPI, RobotReviewer was integrated into EPPI-Reviewer, a systematic review software [27]. Both assessment and consensus occurred within EPPI-Reviewer. In the second arm (RR-web), we used RobotReviewer’s own pilot web solution, http://www.robotreviewer.net. The web solution produces RobotReviewer’s assessments that can be downloaded, but reviewers must conduct their assessments and come to a consensus in a different platform, such as in a Word document. See Table 1.

**Table 1:** Arm descriptions

Procedure	RR-EPPI arm	RR-web arm
Setting up the study	Project leader: - uploaded study files to EPPI-Reviewer - ran RobotReviewer by clicking a button	Project leader: - uploaded study files into RobotReviewer’s website - ran RobotReviewer by clicking a button - downloaded the resulting documents with automated assessments - transferred each document (one per study) to a shorter document with instructions - made the documents available to reviewer pairs
Individual risk of bias assessments (2 reviewers per study)	Researchers assessed risk of bias in EPPI Reviewer.	Researchers wrote their risk of bias assessments into their own copy of the document.
Coming to a consensus	Conducted within the EPPI Reviewer software	Researchers produced a new document

#### Participants and included systematic reviews

We recruited two project leaders of ongoing commissioned systematic reviews (projects A and B), and their six project members. These projects were commissioned by the Norwegian Labour and Welfare Administration and the Norwegian Center for Violence and Traumatic Stress Studies, respectively. Project members had different levels of experience with systematic reviews, and none had previous experience with RobotReviewer. Half of the participants in both projects were newer researchers with two or fewer years of experience in evidence synthesis. The other half were more experienced researchers with nine or more years of experience. Within each project, participants volunteered to take part in the study, and no participants declined. See Table 2 for an overview of participant experience and roles.

**Table 2:** Participants role

Participant	Project	Years of evidence synthesis experience	Dichotomized level of experience	Role
a	A	< 2	Less	Reviewer 1 + Reviewer 2 (b, d)
b	A	< 2	Less	Reviewer 1 + Reviewer 2 (a)
c	A	10+	More	Reviewer 1 + Reviewer 2 (a, d)
d	A	10+	More	Reviewer 1 + Reviewer 2 (c)
e	B	< 2	Less	Reviewer 1 + Reviewer 2 (f)
f	B	10+	More	Reviewer 1 + Reviewer 2 (e)

**Fig. 1:**
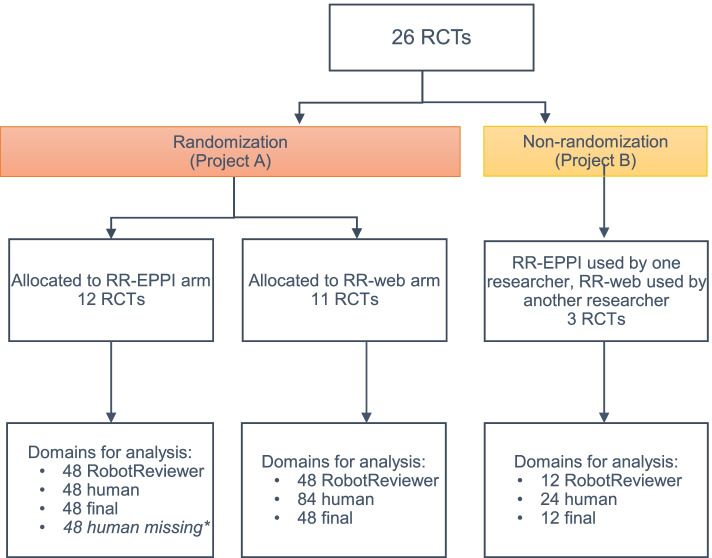
Flowchart of included studies and their domaine assesement available for analysis

In Project A [28], we randomized the included RCTs to RR-EPPI or RR-web. Figure 1 shows the randomization of studies, as well as the unique domains ultimately available for analysis (48 domains were missing due to missing one human’s assessments for twelve studies; four domains per study). For individual assessment and consensus, reviewers tracked time used per study themselves and were instructed to read RobotReviewer’s assessments. Project B [29] contributed 3 RCTs, and we did not randomize these studies. The ML team trainer followed the study set-up procedures (Table 1) for both the RR-Web and RR-EPPI arm, and the two reviewers picked which platform they wanted to use. Researchers in Project B came to consensus of their risk of bias judgement in a Word document, as in the RR-Web arm.

#### Data collection

We collected the following data for the first four Cochrane RoB1 questions of each study: RobotReviewer’s automated assessment, reviewer 1’s assessment, reviewer 2’s assessment, and the final consensus assessment. Each project assessed risk of the bias for one primary outcome. We treated each individual human assessment as its own unit of analysis, which meant there were twice as many individual human assessments than RobotReviewer and final assessments, for the same domain. We asked each reviewer to track their time in an Excel spreadsheet, per RCT and per step (individual assessment and consensus meeting).

To explore acceptability, we emailed reviewers with questions regarding their experiences with RobotReviewer one week after each project completed its risk of bias assessments. After each project’s completion, the ML team presented the accuracy results of the study (Figs. 3 and 4) to the reviewers and projects leaders. PSJJ facilitated group discussion and asked reviewers open-ended questions that began by engaging them with these results. Appendix 1 presents the questions asked. All questions and discussions were in Norwegian, and PSJJ and AEM jointly translated quotations presented in the Results.

### Analysis

#### Accuracy

We measured human corrections to each risk of bias question before consensus, and human corrections to each other, that is, differences between the final assessment after consensus and each individual human assessment made before consensus. We used alluvial diagrams created using https://rawgraphs.io to visualize question-level changes from RobotReviewer assessment, individual human assessment, and final assessment. Each question per study was represented twice in the alluvial diagrams, in order to display the two independent human assessments it received. A RobotReviewer assessment of “high/unclear” was counted as accepted if a subsequent human assessment was “high” or “unclear”.

Humans were able to correct between zero and four questions per study, with zero meaning no corrections were judged necessary, and four meaning a human changed all four risk assessments. Any difference between an individual human assessment and the RobotReviewer assessment was interpreted as a human correction to RobotReviewer. Any difference between the final consensus assessment between the two reviewers (the gold standard) and either of the two individual human assessments was interpreted as a human correction to another human.

We tested for a difference between the distributions of frequency of corrections humans made to RobotReviewer compared to each other using a χ^2^ test, and for relationships between the number of corrections humans made to RobotReviewer assessments and reviewer experience level, reviewer order, and question number (1–4) using appropriate tests (see Results).

#### Time use

We were not able to compare time use of manual assessments versus RobotReviewer-informed assessments, as no arm included manual assessments as a comparison. We are therefore not able to comment on how RobotReviewer could tighten the “synthesis gap” in this review.

#### Acceptability

We collected all answers given via email and all notes taken during group discussions; all answers and discussions were in Norwegian. We attempted to thematically analyze this body of text [30], and to organize according to responses and feedback that were *positive* to RobotReviewer or *negative.* However, *positive* and *negative* soon proved to be insufficient, as many responses combined both elements and expressed ambiguity. We decided to focus on this ambiguity and explore how participants were understanding their relationships to RobotReviewer, as a representation of automation. One researcher thematically sorted the data and summarized it (PSJJ) and two researchers (AEM, HMA) overviewed the sorted data. After coming to agreement, the first researcher who is a Norwegian native speaker translated the qualitative data from Norwegian to English. To make sure that the meaning of the text was not lost in translation, AEM - a native English speaker - double-checked the translations.

## Results

Project A contributed 23 RCTs. Data was missing for the second assessor for twelve studies. Project B contributed three RCTs. For each study, four RobotReviewer assessments were available for analysis, four assessments from the first reviewer, four assessments from the second reviewer, and the final consensus set of four assessments. There were 160 human corrections made to RobotReviewer’s assessments before consensus, and identically 160 human corrections made to each other, that is, differences between the final consensus assessment and each individual human assessment.

### Accuracy

#### 1. Human corrections to RobotReviewer and to human reviewers

**Fig. 2:**
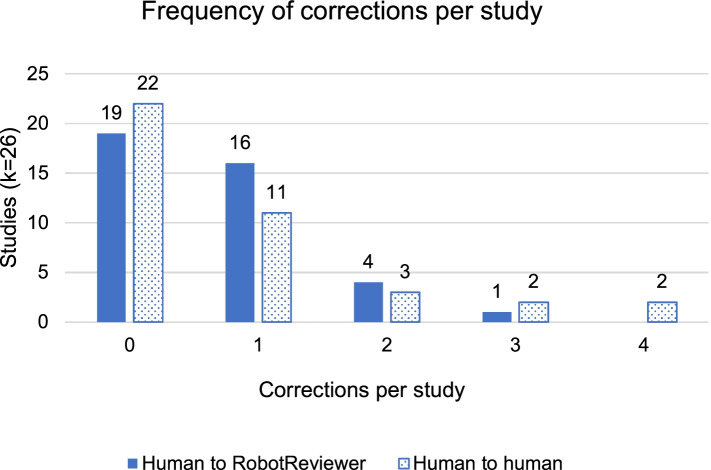
Human corrections to RobotReviewer’s assessments and to other humans’ assessments

There was a statistically significant difference between the distributions of the frequency with which humans corrected RobotReviewer versus one another (χ^2^_12_ = 31.260, *p* < 0.001; see Fig. 2). Humans made the most corrections when they were correcting other human assessments, and rarely made more than two corrections to RobotReviewer. Nevertheless, corrections were the exception, rather than the rule: humans made zero corrections to each other in 55% of studies, compared to zero corrections to RobotReviewer in 48% of studies.

**Fig. 3:**
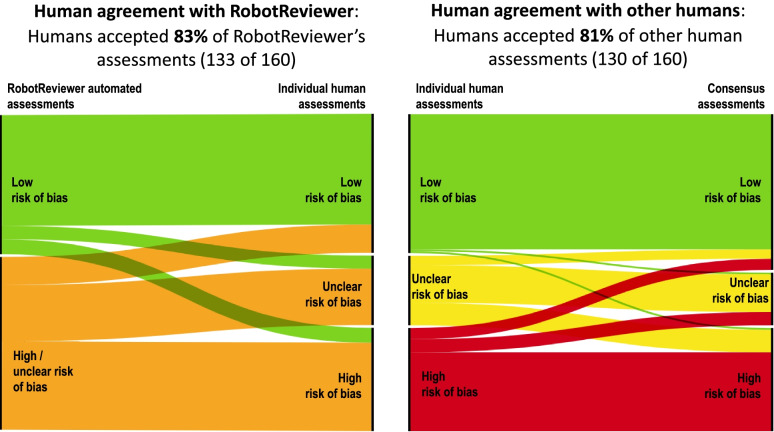
Corrections between individual human assessments and RobotReviewer (left) and between individual human assessments and final consensus (right), in the assessments of all 26 RCTs

Figure 3 shows alluvial plots that illustrate how assessments made by RobotReviewer (left-hand panel) and a human (right-hand panel) were accepted or corrected by humans. The four RobotReviewer assessments and four final consensus assessments received by 14 studies are shown twice in Fig. 3, in order to separately display the two human’s individual assessments per risk of bias question. Humans made 160 unique individual assessments for the 26 studies. For the remaining 12 studies, only one individual human assessment was available per question. RobotReviewer performed as accurately as any one reviewer: humans accepted 83% of RobotReviewer’s assessments (133 of 160), and 81% (130 of 160) of each other’s assessments; risk ratio 1.02 (95% CI 0.92 to 1.13; *p* = 0.33). Note that a RobotReviewer assessment of high/unclear was counted as accepted if a human assessment was “high” or “unclear”.

**Fig. 4:**
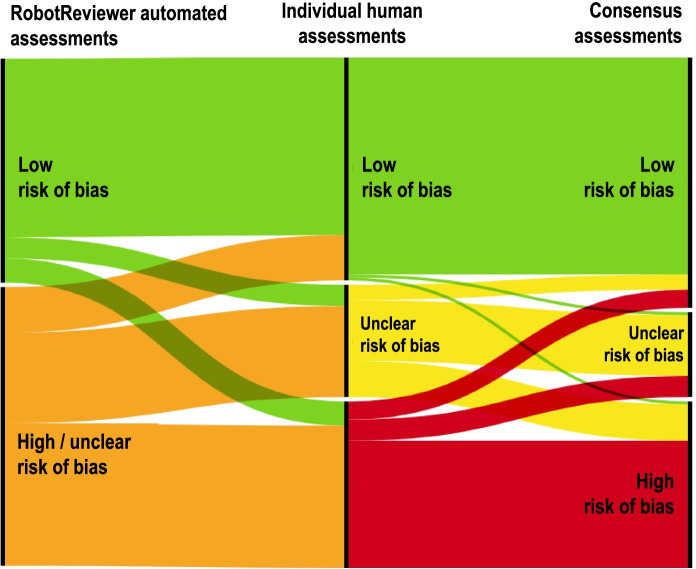
Corrections from RobotReviewer to any one human assessment to the final consensus assessment

Figure 4 below shows an alluvial plot that illustrates how RobotReviewer’s assessments were first corrected by a single human and finally changed during a consensus assessment between the two humans (the gold standard). There was full agreement between RobotReviewer, one human, and the consensus assessment in 79% (126 of 160) of questions.

In 4% (8 of 160) of questions, RobotReviewer under-estimated risk of bias by suggesting a question be answered by *low risk of bias*, with the final consensus assessment being *unclear* (seven questions) or *high* (one question).

#### 2. Question-specific corrections

RobotReviewer’s fourth question – blinding of outcome assessment, in the detection bias domain – was corrected by humans most often: half of human-to-RobotReviewer corrections were made to this question (13 of 27). In contrast, human corrections to each other were evenly distributed among the first three questions (9, 10, and 8, retrospectively, of 31), and humans corrected each other’s assessment to this fourth question least often (5).

#### 3. Associations with corrections

The number of individual corrections made to RobotReviewer was no different for less experienced researchers (median 1) and for more experienced researchers (median 1); Mann-Whitney test U(N_less experienced_ = 22, N_more experienced_ = 18) = 171.5, *z* = − 0.792, *p* = 0.492). Similarly, there was no statistically significant difference between the number of corrections to RobotReviewer made by the first researcher assessing risk of bias for the specific study (median of 1) compared to the second researcher; Mann-Whitney test U(N_first_ = 26, N_second_ = 14) = 180.0, *z* = − 0.62, *p* = 0.967).

We tested these same variables against the number of human corrections made to each other, presented in Fig. 2. There was no statistically significant difference in the number of corrections made to each other (median of 0); Mann-Whitney test U(N_less experienced_ = 22, N_more experienced_ = 18) = 136.5, *z* = − 1.855, *p* = 0.095. However, the first reviewer’s assessments were more likely to become the final assessment; they were corrected less often (median of 0) than the second assessor was corrected (median of 1); Mann-Whitney test U(N_first_ = 26, N_second_ = 14) = 108.0, *z* = − 2.328, *p* = 0.036.

### Time use

Our second research question, assessment with RobotReviewer compared to manual assessment, could not be answered, as both participating project leaders chose to compare two platforms of RobotReviewer against each other.

### Acceptability

We began the one-hour group discussions with a presentation of the quantitative results. The main finding presented was that there was an equal number of corrections of humans to RobotReviewer and to each other. There was little response from participants when asked whether they agreed with this finding. However, the discussion quickly turned to the relationship between RobotReviewer and reviewers. Without being prompted, reviewers reflected on their relationship with automation as represented by RobotReviewer.

All reviewers were positive to using RobotReviewer in future reviews, with a “human-in-the-loop” approach. They did not express discomfort with interacting with an automation tool. The tool’s placement in the current risk of bias assessment procedure was not seen as problematic. However, most were not interested in changing the current procedure from two researchers conducting risk of bias assessment. The following quote demonstrates a complex assessment of the machine-human relationship, in which RobotReviewer is seen as helpful but not trustworthy alone, and human input is ultimately necessary:

“The text was helpful and gave us basic information and helped to focus us visually on a ‘relevant sentence’, but the sentences in the text snippets weren’t always sufficient. We therefore needed to check the article as well… but this didn’t take a long time in well-conducted RCTs”.

Human superiority is emphasized twice: first in supplementing text snippets, and second, indirectly, by calling attention to how quickly humans can conduct this necessary supplementation. This was the only instance in the group discussions that human’s time use was mentioned.

Human assessment was seen as necessary not only to complete the normal risk of bias task but also to reassess RobotReviewer’s assessment, when the automated assessment was obviously incorrect. As one reviewer stated “When RobotReviewer was right, it was spot-on. When it was wrong, it was really off;” this prompted agreement by several reviewers. When reviewers discussed supplementing the standard two-reviewer system with RobotReviewer, they did not frame it as a question of mistrust or of poor performance, but because they did not want to “lose out” on the valuable discussions that occur between two humans when coming to an agreement.

The less experienced reviewer-pair pointed out that disagreements with RobotReviewer stimulated better assessment conversations*:* “We did not always agree with RobotReviewer’s assessment, which might have led us to discuss the risk of bias categories [with each other] more thoroughly.” A common discussion point for this pair was how to assess the fourth question, blinding of outcome assessment, for outcomes extracted from registries, and there was often little agreement between reviewers themselves and between reviewers and the automated assessment.

In general, less experienced reviewers were more positive to RobotReviewer than more experienced reviewers. Most reviewers suggested RobotReviewer could provide additional help within the current practice of two researchers assessing risk of bias and a pedagogical tool for newer researchers. The less experienced reviewers focused on the text snippets provided, saying that these snippets guided them to where in the article they should look to make their own assessments. Two of the newer researchers submitted written feedback together, and wrote, “RobotReviewer is a resource for where to look in the text. The amount of text was also a clue, not a problem; it was most useful on the domain regarding blinding*”*. Even when they did not carry forward this extra text in their own assessments, they found it helpful to have more text to better understand the study context. The fact that they defended extra text as “not a problem” in written feedback provided before discussing in a group, suggests that they had already heard negative feedback from others in the study.

Experienced reviewers did not mention using text snippets as visual cues. Rather, they reacted to what they called “unnecessary information” presented in snippets. What less experienced reviewers appreciated and described as “extra” or a “a clue”, more experienced reviewers described as distracting and unnecessary. The expectation among some experienced reviewers was that the text snippet in its entirety should be relevant and correct; because it was not, RobotReviewer was not deemed helpful. In contrast, less experienced reviewers approached RobotReviewer more flexibly and were less overwhelmed by irrelevant text.

## Discussion

### Main findings

As part of a machine learning (ML) strategy in our evidence synthesis division [20], we implemented a real-time mixed methods study to explore the feasibility of using RobotReviewer within two ongoing systematic reviews. The first notable aspect of this study is that neither of the two project leaders were interested in comparing RobotReviewer to fully manual procedures, indicating a baseline level of interest in automation. This study therefore randomized the RCTs included in the two reviews to two different platforms: RobotReviewer integrated into a systematic review software, or RobotReviewer using the publicly available demonstration website. Quantitatively, humans corrected each other’s assessments slightly more thoroughly than they corrected RobotReviewer’s assessments. Qualitatively, despite RobotReviewer appearing to perform equally as well as any human, reviewers insisted that the tool should not replace one of two humans but should rather be added to the current, resource-intensive, two-human procedure.

RobotReviewer was at least as accurate as any one human, when assessed by another human. In fact, reviewers were more likely to heavily correct each other than RobotReviewer. Hirt et al. [31] also found RobotReviewer’s reliability to be equal to the reliability of a team of eight different reviewers. Studies that were difficult for reviewers to assess, were also difficult for RobotReviewer to assess, when measured by differences between the final assessment and the original human or RobotReviewer assessment. Yet while these results suggest domain-specific difficulties (by both humans and RobotReviewer) rather than automation-specific difficulties, reviewers tended to zero in on only the latter. While participants agreed that “When RobotReviewer […] was wrong, it was really off”, in reality, the opposite was true: humans rejected all or the majority of each other’s assessments in several studies, but never did so for RobotReviewer’s assessments.

Our reviewers reported RobotReviewer to be an acceptable, useful tool. The automated assessments were felt to trigger discussion around risk of bias but were not automatically accepted as correct. Newer reviewers highlighted RobotReviewers usefulness as a pedagogical tool in implementing risk of bias assessments. Our reviewers might have focused more on text snippets because they were judging the accuracy of the automated assessment, and if this is the case, these text snippets provide an important element of transparency to this tool. Even if users do not understand the ML behind RobotReviewer’s assessments, they can try to trace how a piece of text was “interpreted” as an assessment category.

We could not answer our third research question quantitatively because neither project leader was interested in using manual procedures as a comparison. The project leaders’ baseline level of interest in automation was not reflected among the reviewers on their team; an important reminder that top-down support for automation does not necessarily trickle down. While participants did not dwell on time spent or saved, their experiences of RobotReviewer’s utility are likely related to their overall experience with risk of bias assessment. Experienced reviewers may have found RobotReviewer less helpful because their experience enabled them to identify relevant text faster (without needing the aid of automatically extracted text snippets). They may also have had more of an interest, than newer reviewers, in defending the scope of their responsibilities within a review.

We found that adding RobotReview to standard practice of two reviewers is feasible when using Cochrane’s RoB1 tool. A separate question is whether standard practice itself should be changed to more fully rely on RobotReviewer, for example by using only one human plus RobotReviewer. To answer this question, a future study should compare RobotReviewer against the standard manual procedure.

Based on this work, we anticipate that RobotReviewer, even added to a two-reviewer process, will save time compared to a fully manual two-reviewer process, particularly when one or both reviewers are less experienced. For reviews utilizing RoB1, we recommend further use of RobotReviewer either added to a two-reviewer process or used in tandem with only one experienced reviewer: our findings suggest that newer reviewers are likely to benefit from using RobotReviewer; we do not anticipate a loss in quality; and provision of text snippets provides a degree of transparency to the ML process behind RobotReviewer, which may lead to increased trust in reviews that have used this automated tool. While there is potential for RobotReviewer to support more standardized assessments *across* research teams and institutions, reviewers need time to build trust in this tool and in accepting changes to established workflows.

The next version of the tool, RoB2 [32], has become more widespread since we conducted this study, for which RobotReviewer is not (yet) adapted. We anticipate that a portion of future reviews may still find it reasonable to proceed with RoB1, for example review updates that opt to use the same risk of bias tool as the original review, or large reviews with time constraints, as time use does seem to be increasing as the tool becomes more refined [10, 33]. However, even if all use of RoB1 were to cease in the near future, we hope the more qualitative findings of this study will remain informative: reviewers’ ambivalence to automation reduces acceptance of a tool, and quantitative evidence of performance is not necessarily sufficient to overcome this ambivalence. Institutions will need to learn how to continually identify and address ambivalence and prepare reviewers to be agile with respect to embracing updated methods (such as improved risk of bias tools) and to fluctuating human roles and activities thanks to automation.

### Methodological considerations

This study has several limitations. We operationalized feasibility as accuracy, relative resource use, and acceptability. We did not systematically measure feasibility according to the structural factors that influence adoption of an innovation, such as administrative and technical factors, or design aspects that affect the user’s experience. A major strength and novelty of this study is its real-time implementation within two reviews and its user-driven design. While it is certainly interesting that neither project leader wished to include a manual arm, the consequence was that we were therefore not able to estimate the relative time use of RobotReviewer against manual practices. Neither did we query participants as to whether they perceived RobotReviewer to reduce or increase the time required of them. Another weakness is the small sample size of only 26 RCTs. We encourage other researchers to repeat our evaluation with a larger sample of studies, and a systematic review to synthesize results across these future studies. We wrote an evaluation plan, in Norwegian, for this study for internal use (we did not publish a formal protocol), and we did not deviate from our original research questions. Finally, given that RobotReviewer assessments were dichotomized but human assessments were conducted in three risk of bias categories, human assessments will be more likely to be corrected than the slightly simpler RobotReviewer assessments.

Related to this study’s user-driven design is the close working relationship of the study administration (AEM and PSJJ) with participants. AEM and PSJJ, in their capacity as ML team lead and member, respectively, were tasked with implementing and encouraging the use of ML functions by their colleagues. AEM in particular worked closely with project leaders and members to build enthusiasm for this study. It is quite possible that social desirability bias was present in discussions of acceptability; participants may have withheld more critical perspectives in the group discussion to be polite. However, while politeness could presumably bias the opinions they shared, it could not bias the number of corrections.

We suggest that the human (and social) element cannot and should not be stripped from ML. Whether it is a software provider presenting a software, an IT employee helping a reviewer to use a tool, or an enthusiastic project leader teaching their team, ML will always involve humans at some point within a larger process. Rather than see human involvement as a source of bias for our results, we instead highlight human involvement as an important aspect in ML roll-out and trust-building within evidence syntheses.

## Conclusion

RobotReviewer performed as accurately as any of the researchers in a two-human risk of bias assessment of systematic reviews. At the same time, implementing an automation tool to assist a previously manual process requires more training than we provided, and probing reviewers’ expectations before beginning. Newer reviewers may be more open to automation in preforming a task that they are not as experienced in. Targeting newer reviewers could be an important element in building support for these tools. In this study, reviewers acknowledged the utility of RobotReviewer, but saw human-to-human interaction and discussion as too important to remove from risk of bias assessment.

## Supplementary Information

**Additional file 1: Appendix 1.** Interview guide.

## Data Availability

The datasets generated and/or analyzed during the current study are not publicly available due as they are part of in-house products, but are available from the corresponding author on reasonable request.

## Abbreviations

CDSR: Cochrane Database of Systematic Reviews
GRADE: Grading of Recommendations, Assessment, Development and Evaluations
ML: Machine learning
NIPH: Norwegian Institute of Public Health
PDF: Portable Document Format
RCT: Randomized controlled trial
RoB1: Cochrane Collaboration’s tool for assessing risk of bias version 1
RoB2: Cochrane Collaboration’s tool for assessing risk of bias version 2
RR-EPPI: RobotReviewer integrated into the EPPI-Reviewer software
RR-web: RobotReviewer’s own pilot web solution
