# Supplementary material for: Automating risk of bias assessment in systematic reviews: a real-time mixed methods comparison of human researchers to a machine learning system
Source: BMC Med Res Methodol. 2022 Jun 8;22:167. doi: 10.1186/s12874-022-01649-y (PMC9174024; doi:10.1186/s12874-022-01649-y)
Supplement: Supplementary file 1 — Additional file 1: Appendix 1. Interview guide. [file 12874_2022_1649_MOESM1_ESM.docx]

Appendix 1: interview guide

| **Acceptability questions sent to each reviewer by email (n=6 participants)** | **Acceptability prompts in the group discussions (k=2 discussions)** |
| --- | --- |
| - How was it to assess as reviewer number 1, if applicable? (with access to RobotReviewer but not to your reviewer partner’s assessment) - How was it to do the assessment as reviewer nr 2? (with access to both the first reviewer’s assessment and RobotReviewer assessments?) - How was it to reconcile human assessments with access to RobotReviewer? - Any other thoughts or feedback about future use of RobotReviewer? | - What did you think about the results of the study? - Is there anything we should have done differently? - What do you think about future use of RobotReviewer? |
